# Post-Transplant Surveillance and Management of Chronic Active Antibody-Mediated Rejection in Renal Transplant Patients in Europe

**DOI:** 10.3389/ti.2023.11381

**Published:** 2023-07-17

**Authors:** Lionel P. E. Rostaing, Georg A. Böhmig, Ben Gibbons, Muhammed Mahdi Taqi

**Affiliations:** ^1^ Service de Néphrologie, Hémodialyse, Aphérèses et Transplantation Rénale, CHU Grenoble-Alpes, Grenoble, France; ^2^ Clinical Department of Nephrology and Dialysis, University Clinic for Internal Medicine III, Medical University of Vienna, Vienna, Austria; ^3^ Bryter Inc., New York, NY, United States

**Keywords:** kidney transplant, antibody-mediated rejection, chronic active, post-transplant surveillance, CABMR

## Abstract

Antibody mediated rejection (ABMR) is the leading cause of immune-related allograft failure following kidney transplantation. Chronic active ABMR (CABMR) typically occurs after one-year post-transplant and is the most common cause of late allograft failure. This study was designed to assess common practices in Europe for post-transplant surveillance 1 year after kidney transplant, as well as the diagnosis and management of CABMR. A 15-minute online survey with 58 multiple choice or open-ended questions was completed by EU transplant nephrologists, transplant surgeons and nephrologists. Survey topics included patient caseloads, post-transplant routine screening and treatment of CABMR. The results indicated that observing clinical measures of graft function form the cornerstone of post-transplant surveillance. This may be suboptimal, leading to late diagnoses and untreatable disease. Indeed, less than half of patients who develop CABMR receive treatment beyond optimization of immune suppression. This is attributable to not only late diagnoses, but also a lack of proven efficacious therapies. Intravenous Immunoglobulin (IVIG), steroid pulse and apheresis are prescribed by the majority to treat CABMR. While biologics can feature as part of treatment, there is no single agent that is being used by more than half of physicians.

## Graphical Abstract

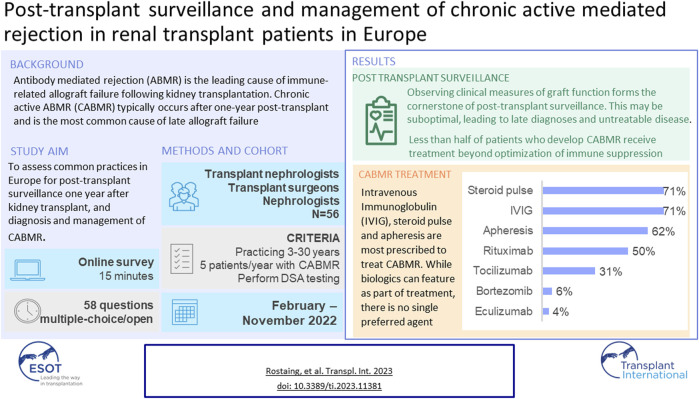

## Introduction

Antibody mediated rejection (ABMR) is the leading cause of immune-related allograft failure following kidney transplantation [1, 2]. Although the pathophysiological pathways that give rise to ABMR are yet to be fully elucidated, it is understood that B cell and plasma cell activation leads to generation of donor-specific antibodies (DSAs), which bind to human leukocyte antigen (HLA) or non-HLA molecules expressed on endothelial cells within the kidney allograft [1, 3, 4]. Chronic active antibody mediated rejection (CABMR) typically occurs after one-year post-transplant and manifests as a slower decline in graft function than acute ABMR. Risk for negative outcomes is higher for those who develop CABMR, including graft loss or death [4].

CABMR is characterized by evidence of both chronic disease (interstitial fibrosis, tubular atrophy, transplant glomerulopathy) and active disease components (glomerulitis, peritubular capillaritis) [5]. It is the leading cause of late allograft failure; within 2 years of diagnosis, over 75% of those with CABMR lose their graft [6].

Maintenance immunosuppression starting prior to or immediately post-transplant is required in order to prevent immune-related graft injury (including CABMR). Despite maintenance immunosuppression, CABMR continues to be a challenge. The reasons why current immunosuppression protocols fail to prevent the development of CABMR are not yet fully understood, however contributing factors have been identified: patient non-adherence with immunosuppression [2, 7], and insufficient immunosuppression [2].

Post-surveillance monitoring is crucial to ensure optimized maintenance immunosuppression and to detect signs of graft dysfunction. While consensus guidelines on the management of patients post-transplant exist [8], these have not been updated to discuss recent advancements in testing (DSA testing and cell-free DNA testing) and the case for protocol biopsies. Clinical measures of graft function (eGFR, serum creatinine, proteinuria), monitoring DSAs, and biopsies are typically used for surveillance. In recent studies, donor-derived cell-free DNA in peripheral blood has gained interest as a potential non-invasive biomarker following demonstration of ABMR association with serum concentrations of donor-derived cell-free DNA [7].

Treatment options for CABMR are limited beyond optimization of immune suppression. A relative lack of evidence for specific treatments means that there is no current consensus on CABMR management in Europe. IVIG, apheresis and corticosteroids are widely used treatment options. There is also evidence to support the use of biologics. These include B-cell targeting agents (e.g., rituximab [9, 10]), complement targeting agents (e.g., eculizumab), and more recently, agents targeting IL-6 pathways, (e.g., tocilizumab [11]).

This research was designed to assess common practices in Europe for post-transplant monitoring of patients receiving a renal allograft 1 year after transplant. Focus was placed on monitoring for CABMR, and how patients with CABMR are typically managed once diagnosed.

## Materials and Methods

52 transplant nephrologists, nephrologists and transplant surgeons were recruited by email invitation through targeted lists provided by ESOT, then screened and profiled to ensure a good representation of the European market was achieved. Physicians must have been in practice for 3–30 years, see a minimum of 5 patients/year with CABMR and perform DSA testing post-transplant to qualify. In addition to study-specific screening criteria, respondents were screened to ensure that they are not affiliated with any industry partners. A full breakdown of sample demographics is shown in Table 1. Physicians completed a 15-minute online survey with 58 multiple choice or open-ended questions grouped into sections: patient caseloads, post-transplant routine screening, treatment of CABMR and demographic questions.

**TABLE 1:** Table showing respondent profile breakdown (N = 52).

		Total (*n* = )
All respondents		52
Specialty	Transplant nephrologist	41
	Nephrologist	9
	Transplant surgeon	2
Gender	Male	29
	Female	20
	Prefer not to say	2
Length of time in practice	Less than 3 years	1
	3–10 years	13
	11–20 years	17
	21+	21
Hospital type	Teaching/university hospital	46
	General hospital	4
	Private hospital	1
Number of renal transplant patients followed up with per year	5–50 patients	6
	51–100 patients	23
	101+ patients	23
Country of practice	Italy	10
	France	9
	Spain	5
	United Kingdom	4
	Netherlands	4
	Germany	3
	Greece	3
	Belgium	2
	Croatia	2
	Poland	2
	Portugal	2
	Austria	1
	Czech Republic	1
	Sweden	1
	Switzerland	1
	Bosnia and Herzegovina	1
	Montenegro	1

Data was aggregated and described using the mean and range. In order to determine whether findings were statistically significant, we used *t*-test for parametric data and chi-squared for non-parametric data. A *p*-value ≤0.05 was considered statistically significant.

## Results

### Post-Transplant Monitoring in the 1st Year Post-Transplant

Proteinuria and serum creatinine are tested frequently in the first-year post-transplant: 88% assess creatinine and 79% assess proteinuria every 1–3 months, rising to 100% assessing creatinine and proteinuria every 1–6 months.

Frequency of DSA testing was found to vary by patient type. Physicians are more likely to routinely assess pre-sensitized patients for *de novo* DSA (81% of physicians report doing so at least once a year) than patients who are not pre-sensitized (67% assess these patients at least once a year) (t(55) = −2.00, *p* = 0.03). 27% and 19% indicate that they do not routinely test for *de novo* DSA in non pre-sensitized and pre-sensitized patients (respectively) after the 1^st^ year post-transplant. The One Lambda Luminex^®^ platform assay is the most used DSA testing method—utilized by 69% of physicians—followed by the Immucor Luminex^®^ platform assay (29%).

Surveillance (protocol) biopsies are not routinely performed by physicians. Only 27% perform them routinely at 6–12 months post-transplant and significantly less perform them routinely after 1 year (15%, t(51) = −2.58, *p* = 0.05).

Use of cell free DNA testing is not widespread with only 13% of physicians using the test for a small portion of their patients.

**FIGURE 1:**
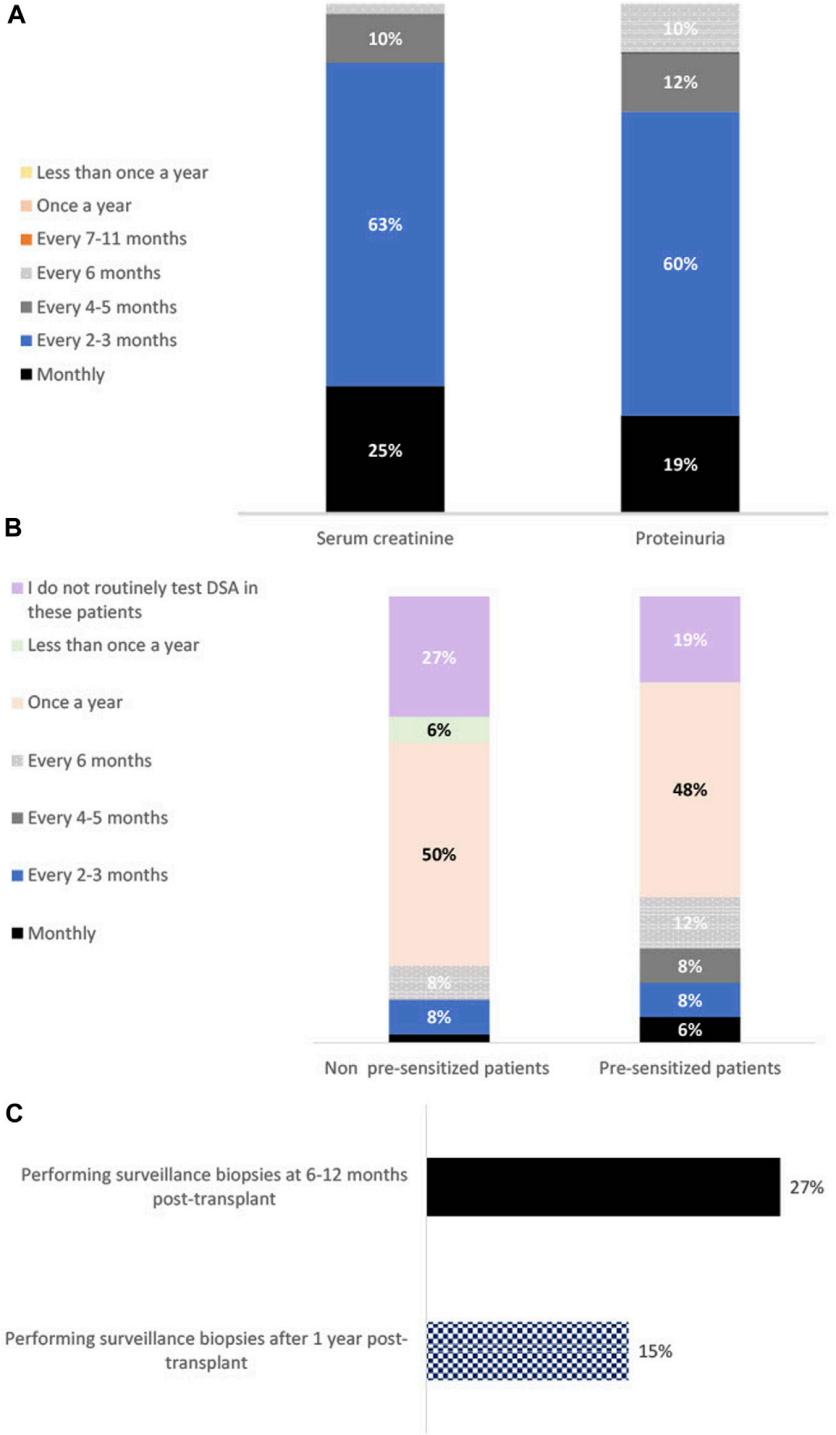
Bar charts showing percentage using each surveillance method 1 year post-transplant, and their frequency of usage. Panel **(A)** shows the percentage of physicians testing for serum creatinine and proteinuria at each time interval (*n* = 52). Panel **(B)** shows the percentage of physicians carrying out DSA testing at each time interval in patients that are not sensitized (determined by lack of detectable DSA) at transplantation and those that are pre-sensitized at transplantation (*n* = 52). Panel **(C)** shows the percentage of physicians using surveillance kidney allograft biopsies at 6–12 months post-transplant vs. 1 year post transplant (*n* = 52).

See Figure 1 for a summary of post-surveillance tests performed in the 1st year post-transplant, and their frequency.

### Prevention and Treatment of CABMR

**FIGURE 2:**
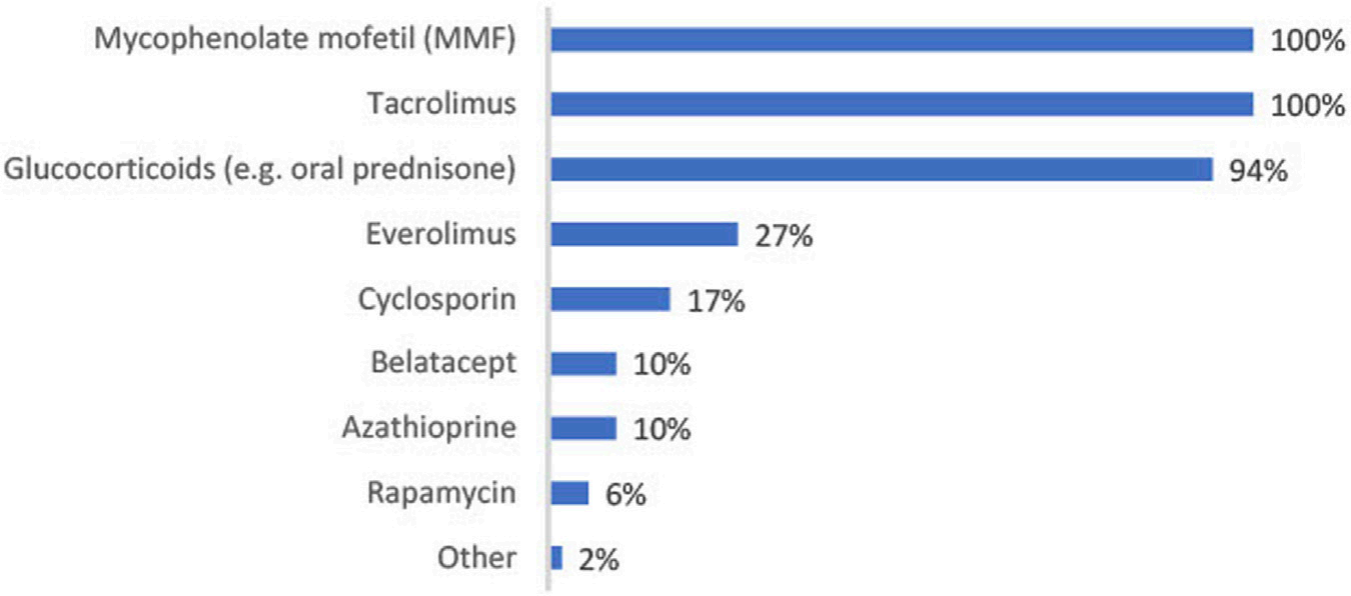
Bar chart showing percentage of physicians using each maintenance immunosuppressive treatment post-transplant (*n* = 52).

Mycophenolate mofetil (MMF) tacrolimus and glucocorticoids are the maintenance immunosuppressive treatments primarily used to prevent immune-mediated rejection. MMF and tacrolimus are used by the entire sample (100%), and 94% use glucocorticoids (see Figure 2 for other maintenance treatments used).

**FIGURE 3:**
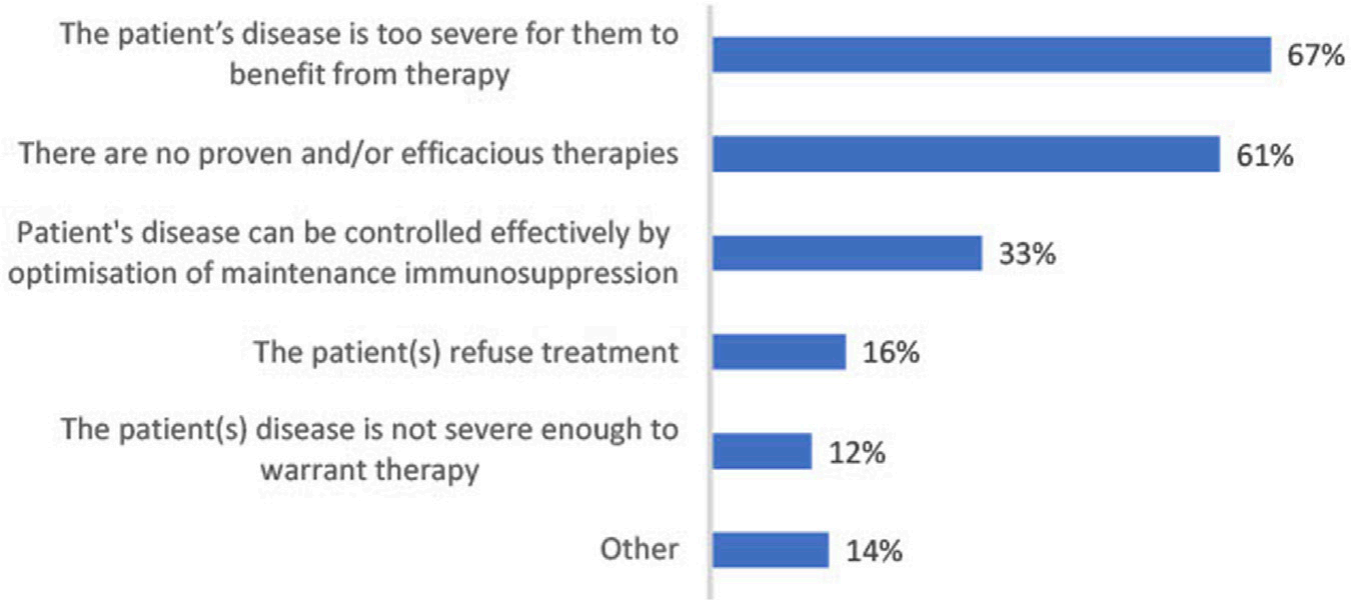
Bar chart showing most frequent reasons for not prescribing further treatment for CABMR beyond optimization of immune suppression given by those who said at least some of their patients receive no treatment except for maintenance immunosuppression (*n* = 49).

Beyond optimization of immune suppression, additional treatment is not received by around half (52%) of all CABMR patients; the reasons why are listed in Figure 3. The advanced severity of disease is the primary reason for this (67%). Other factors include: a lack of proven/efficacious therapies (61%), the belief that disease can be controlled with immunosuppression optimization alone (33%), patient refusal of treatment (16%) and disease severity not warranting further treatment (12%). Of the patients who do not receive treatment beyond optimization of immunosuppression, on average 57% achieve adequate disease control.

**FIGURE 4:**
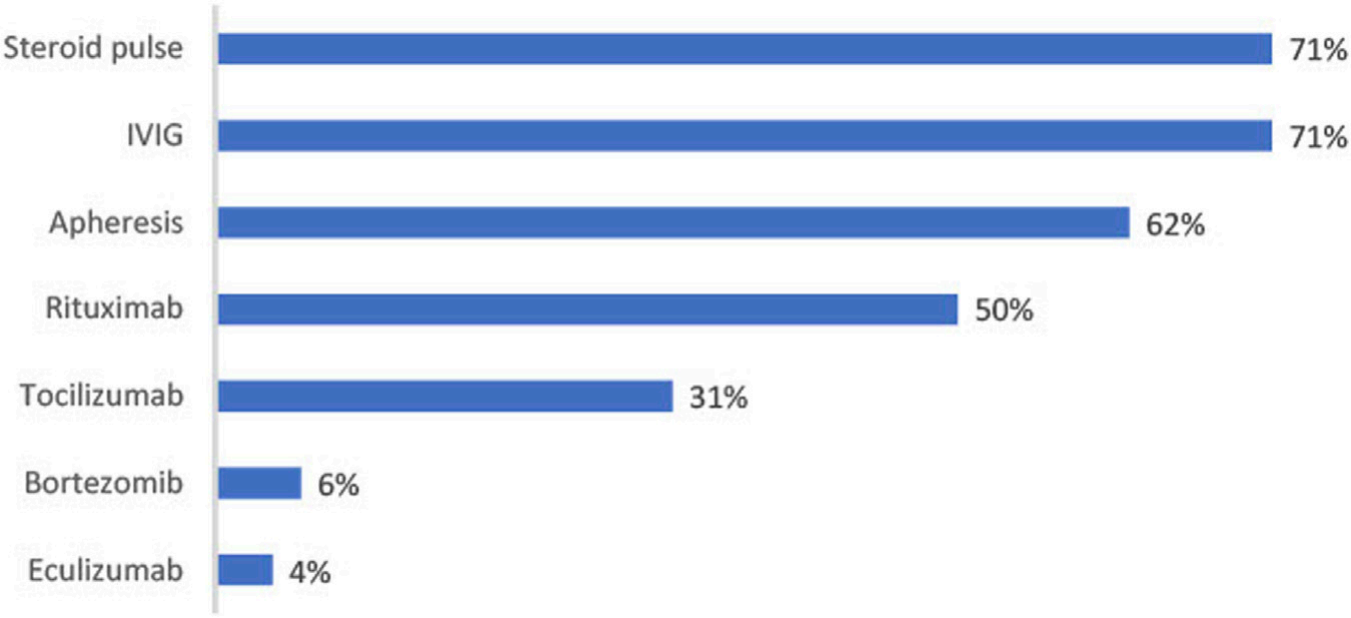
Figure shows percentage of physicians prescribing each treatment for CABMR (*n* = 52).

The current therapies used for the treatment of CABMR are illustrated in Figure 4. IVIG, steroid pulse and apheresis are most commonly used with 71%, 71% and 62% respectively using these therapies. Of those prescribing steroid pulse treatment, 92% prescribe between 5 mg/kg and 10 mg/kg. An average of 3 doses (SD = 1.63) are prescribed. Of those prescribing IVIg, 74% of physicians prescribe a dosage of 1 mg/kg or less. An average of 4 doses (SD = 4.13) are prescribed.

Biologics are not used as frequently; rituximab is the most widely used (50% prescribe this treatment), followed by tocilizumab (31%), bortezomib (6%) and eculizumab (4%).

## Discussion

### Post-Transplant Surveillance

Previously, poorly HLA-matched transplants and transplantation of poorer quality organs [12] were too high risk for transplantation. Recent advancements in transplant science, including preservation and tolerance techniques, are allowing for transplantation of these suboptimal organs. These types of transplants are at greater risk of post-transplant complications [12]; therefore, the need for effective surveillance post-transplant has increased.

Our findings indicate that post-transplant surveillance in Europe centers on clinical measures of graft function (proteinuria and creatinine levels) and, in most cases, testing for *de novo* DSA (dnDSA). Risk factors for graft failure were identified as proteinuria and increased creatinine [13, 14]. Detection of dnDSA is considered both a marker and a contributor of ongoing alloimmunity; this is evidenced by an increased rate of estimated glomerular filtration rate (eGFR) decline even before the detection of dnDSA, followed by an accelerated decline after detection of dnDSA [15].

In our introduction we alluded to a lack of guidance regarding surveillance assessment of DSA levels; this is reflected in how testing is implemented in clinical practice. Likelihood of testing and frequency of testing is variable after the first-year post-transplant and is influenced by patient type. More physicians are testing sensitized patients than non-sensitized patients. Indeed, those with pre-existing DSAs are at greater risk for CABMR than patients without DSAs at transplantation [4, 16].

Proteinuria, creatinine and DSA testing appear to be the primary methods for detecting CABMR, although this may be suboptimal. Over half of patients do not receive treatment beyond optimized maintenance immunosuppression; in most cases, this is a result of their disease being too severe to benefit from treatment. Proteinuria and creatinine testing only indicate broad graft function and are not sufficient markers to diagnose subclinical antibody mediated rejection or CABMR alone. eGFR, serum creatinine, or proteinuria levels are not noticeably impacted by subclinical ABMR until extensive morphological damage has occurred [17]. An irreversible loss of function may be experienced by patients before they can be treated for any immune-related graft issues [18]. Additionally, the importance of DSA testing is still a topic of debate, as histologic changes consistent with AMR can still be observed in those with no detectable DSA [15, 19].

Currently, biopsies are the most accurate way to evaluate graft health by identifying two main types of lesions: lesions related to tissue damage and function and lesions related to immune suppression. For CABMR, biopsies are the only way to obtain a definitive diagnosis. Additionally, biopsies are the only accurate method of detecting subclinical rejection, which left unaddressed can lead to loss of graft function and/or total graft loss [15, 17]. Protocol biopsies at 3 months post-transplant can improve 5-year transplant survival rates according to recent findings [18].

Despite the unmatched diagnostic value that biopsies provide, physicians may be reluctant to perform them without specific cause. In the present study, surveillance biopsies at 6–12 months post-transplant are routinely performed by only 27% of physicians, decreasing to 15% performing them one-year post-transplant. This reluctance to perform protocol biopsies may be because their risk-benefit is still unclear [18, 20, 21]. A lack of proven treatments in the category may be leading physicians to feel there is no merit in conducting routine invasive procedures.

Donor-derived cell free DNA testing is of increasing interest as a potential biomarker for CABMR [7, 22] due to its non-invasive nature and the potential to facilitate earlier treatment by detecting subclinical allograft injury. Currently, cell free DNA testing is used by only 13% of physicians surveyed. While the efficacy of cell free DNA testing as a biomarker for ABMR continues to be shown by growing evidence [22, 23], it remains to be seen what role it will play in the future of post-transplant surveillance.

### Treating CABMR

Plasma exchange, IVIG, and steroids for treatment, with the possible addition of rituximab in the setting of dnDSA, were all recommended in recent consensus guidelines for the treatment of CABMR [24]. Our findings are consistent with these guidelines. IVIG, steroids pulses and apheresis are being prescribed by the majority of physicians. Rituximab is being prescribed by half of physicians, and this might be due to a lack of evidence of efficacy and an increase in risk of pneumonia associated with its usage (when combined with IVIg and steroids vs. IVIg and steroids alone) [25].

Despite some evidence for complement targeting treatments for CABMR, optimal regimens have not yet been identified. The level of improvement seen when using these additional agents has not been clinically significant enough to substantially change the treatment paradigm. Further research is needed to determine whether the benefit seen is patient subtype specific, and to facilitate personalization of treatment protocols.

IL-6 targeting strategies for the treatment of CABMR [11] are receiving growing interest due to the role of IL-6 in inflammatory processes and the maturation and activation of T cells, B cells and plasma cells [11]. There is preliminary evidence to support the use of the IL-6R targeting agent tocilizumab in the desensitization of patients with pre-existing DSA prior to transplant and the treatment of CABMR [26], however further randomized controlled trials are required to support these findings. Our study found that 31% of surveyed physicians are prescribing it in the CABMR setting despite the lack of evidence from randomized and controlled trials.

Ultimately, while physicians appear to be aligned on the usage of steroids, apheresis and IVIg for CABMR, further investigation is required for consensus on biologics. IL-6 targeting agents have potential; clazakizumab, an anti-IL6 targeting agent, was assessed in a recent phase 2 study, and evidence was found for modulation of DSA, stabilization of glomerular filtration rate (GFR), and a manageable safety profile [27]. The phase 3 IMAGINE trial assessing the efficacy and safety of clazakizumab is currently ongoing. The unmet need for proven efficacious therapies could be addressed by positive outcomes.

### Study Limitations

A limitation of the present study is that a low proportion of responding physicians are based in high volume centers. Fewer CABMR patients are treated at low volume centers, and physicians employed there have less experience in treating this relatively rare patient type. Subsequently, findings may not be generalizable to how the majority of CABMR patients are treated.

The results may not accurately represent current practices within countries due to the small number of physicians that responded from each country; however, the inclusion of respondents from a range of over 15 European countries provides an understanding of attitudes towards post-transplant surveillance and CABMR treatment across the continent.

## Conclusion

Clinical measures (proteinuria and creatinine levels) of graft dysfunction and DSA testing are currently the cornerstone of post-transplant surveillance. While there is evidence to support their usage, late diagnoses and consequently poor treatment outcomes may be caused by their inability to detect subclinical signs of rejection. Earlier detection of CABMR could occur through surveillance biopsies, but they are not widely used. Cell-free DNA testing is also still in its infancy. When CABMR develops, around half of patients receive treatment beyond maintenance immunosuppression. This is attributable to late-stage diagnoses but also a lack of effective therapies. IVIG, apheresis and steroids are the main treatments prescribed by physicians to treat CABMR, with half prescribing rituximab. Other biologics may be prescribed, but a lack of sufficient evidence is likely limiting their usage.

## Data Availability

The original contributions presented in the study are included in the article/supplementary material, further inquiries can be directed to the corresponding authors.
